# Mosquito-based xenosurveillance reveals circulation of *Anaplasma* and *Neoehrlichia* in livestock at a human–wildlife interface in Kenya

**DOI:** 10.1186/s13071-026-07510-1

**Published:** 2026-06-19

**Authors:** Dennis Getange, Samson Mukaratirwa, Oscar Esibi, Epaphrus Yuko, James Kabii, Rua Khogali, Jandouwe Villinger

**Affiliations:** 1 International Centre of Insect Physiology and Ecology (icipe), P.O. Box 30772-00100, Nairobi, Kenya; 2 School of Agriculture and Science, Westville Campus, University of KwaZulu-Natal, Private Bag X54001, Durban, 4000 South Africa; 3 One Health Centre for Zoonoses and Tropical Veterinary Medicine, Ross University School of Veterinary Medicine, West Indies, Basseterre, St Kitts and Nevis; 4 Department of Veterinary Tropical Diseases, University of Pretoria, Private Bag 20, Pretoria, 0028 South Africa; 5 Department of Parasitology, Faculty of Veterinary Medicine, University of Khartoum, P.O. Box 32, Khartoum North, Sudan; 6 Department of Medicine and Animal Production, University of Naples Federico II, Naples, Italy

**Keywords:** Xenosurveillance, Mosquitoes, Tick-borne pathogens, Vector-borne pathogens, *Anaplasma* spp., *Neoehrlichia*, *Candidatus* Neoehrlichia mikurensis

## Abstract

**Background:**

Xenosurveillance, which uses blood-feeding insects as biological samplers, is an emerging non-invasive approach for monitoring pathogens circulating among humans, livestock, and wildlife. However, its application to livestock-associated bacterial pathogens at human–animal–wildlife interfaces remains underexplored. We investigated whether mosquito blood meals could be used to detect tick-borne bacterial pathogens circulating in livestock in Kenya.

**Methods:**

We collected 4673 mosquitoes, belonging to *Culex*, *Anopheles*, *Aedes*, *Mansonia*, and *Coquillettidia* genera around livestock enclosures in Kajiado and Naivasha counties, Kenya, using CO₂-baited CDC miniature light traps. Traps were selected to maximise species diversity, and as light traps capture fewer engorged mosquitoes than resting traps, only 56 blood-fed individuals (1.2%) were collected and processed as whole-specimen homogenates and analysed for vertebrate blood-meal sources using *cytochrome b* sequencing. In total, 303 mosquito pools, including blood-fed individuals processed as single-mosquito pools, were screened for *Anaplasma*, *Ehrlichia*, *Rickettsia, Theileria,* and *Babesia* using PCR–high-resolution melting analysis and confirmatory sequencing.

**Results:**

All pathogen-positive detections were exclusively from blood-fed individuals. We detected *Anaplasma marginale* in *Culex pipiens* (1/150; 0.7%) and *Aedes hirsutus* (2/11; 18.2%), *Anaplasma* sp. in *Cx. pipiens* (2/150; 1.4%) and *Ae. hirsutus* (1/11; 9.1%), and *Candidatus* Neoehrlichia mikurensis in *Mansonia africana* (2/50; 4%). Pathogen detections showed strong host concordance, where *A. marginale* was associated with cattle-derived blood meals and *Anaplasma* sp. with goat-derived blood meals, while *Candidatus* Neoehrlichia mikurensis was detected in *Mn. africana* that had fed on cattle and on a host that could not be determined.

**Conclusions:**

Our results provide preliminary evidence that mosquito-based xenosurveillance can detect tick-borne bacterial pathogens circulating in livestock at human–wildlife interfaces in Kenya. The strong concordance between pathogen identity and vertebrate host in blood-fed mosquitoes supports the biological plausibility of this approach. Notably, detection of *Ca.* Neoehrlichia mikurensis represents the first report of this zoonotic pathogen in mosquitoes in Africa, highlighting the One Health relevance of xenosurveillance in identifying settings, where pathogen circulation and cross-interface feeding coincide. Mosquito-derived sampling has potential to complement existing surveillance tools in agro-pastoral systems, where direct host sampling is difficult or costly.

**Graphical Abstract:**

**Figure:**
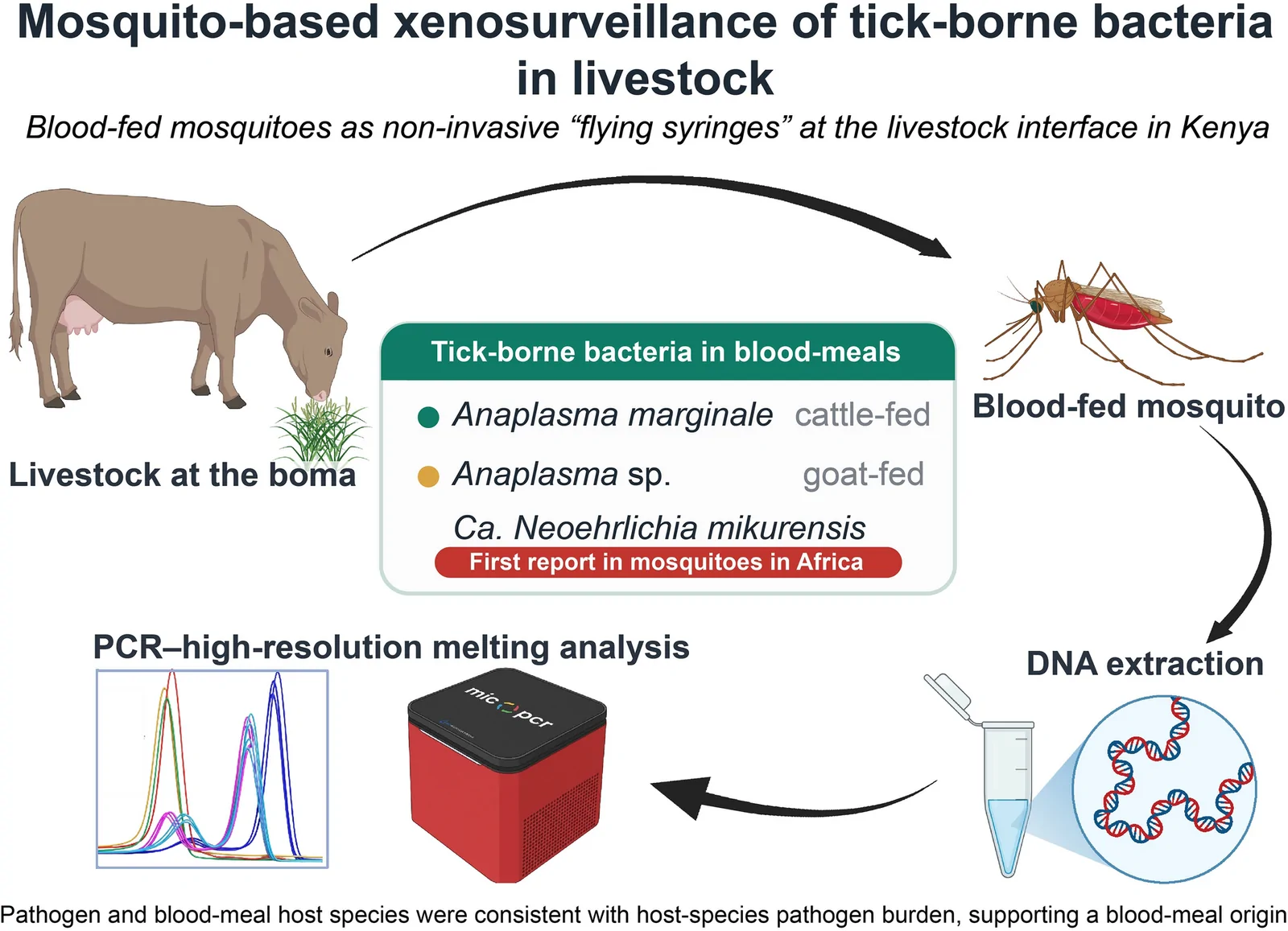

## Background

Over the recent years, the emergence and re-emergence of vector-borne infectious diseases have increased significantly resulting in substantial global economic and public health burdens [1]. This trend is driven largely by ecological, demographic, and climatic shifts that increase interactions among humans, livestock, and wildlife [2, 3]. An estimated 60% of emerging infectious diseases are zoonotic, and more than two-thirds of these originate in wildlife reservoirs [3, 4]. Although viral pathogens constitute a major proportion of these threats, bacterial and protozoan zoonoses, particularly those transmitted by arthropod vectors, remain critically important to both veterinary and human health [5]. Species within the *Anaplasma*, *Ehrlichia*, *Rickettsia*, *Babesia*, and *Theileria* genera circulate in complex enzootic cycles involving domestic animals, wildlife, and hematophagous arthropods [6–8]. These pathogens can cause debilitating diseases, such as anaplasmosis, ehrlichiosis, and theileriosis, leading to significant livestock morbidity and economic losses across tropical and subtropical regions [8]. Therefore, the development of innovative surveillance tools capable of detecting diverse vector-borne pathogens is essential for strengthening early warning systems and improving integrated disease control.

While ticks remain the primary vectors of many bacterial pathogens affecting livestock, growing evidence indicates that non-tick hematophagous arthropods, including mosquitoes, Hippoboscids, biting midges, and stable flies, can occasionally harbour pathogen DNA acquired through blood meals from infected hosts [9–11]. This has renewed interest in their potential roles in mechanical transmission and in xenosurveillance-based pathogen monitoring. Notably, recent experimental work demonstrated that the camel-specific ked *Hippobosca camelina* can transmit *Candidatus* Anaplasma camelii to laboratory mice, indicating the potential role of non-tick blood-feeding flies in the ecology of *Anaplasma* spp. transmission [12]. Although mosquitoes are not biological vectors for these bacteria, their ability to carry detectable pathogen DNA offers opportunities for pathogen monitoring through molecular surveillance approaches. Moreover, mosquitoes already serve as major vectors of diverse protozoan, viral, and filarial pathogens affecting humans, livestock, and wildlife [13, 14], highlighting their global epidemiological importance. Understanding cross-vector interactions at livestock–vector interfaces is, therefore, essential for unravelling the complex ecological networks that shape pathogen transmission in agro-ecological systems.

Use of blood-feeding arthropods as “biological syringes” for detecting vertebrate pathogens, termed xenosurveillance or invertebrate-derived DNA (iDNA) screening, has achieved considerable momentum in recent years [15]. This non-invasive method is based on the analysis of host selection and host preferences of hematophagous arthropods, achieved through the molecular identification of their blood meals [16]. By capitalising on the broad feeding ranges of mosquitoes and other hematophagous taxa, xenosurveillance enables these insects to serve as natural samplers of vertebrate communities, particularly in regions, where conventional wildlife or livestock sampling is logistically difficult [15, 17]. The approach has been successfully applied to detect a wide range of pathogens, including arboviruses, malaria parasites, filarial nematodes, and non-vector-borne agents such as influenza virus and Epstein–Barr virus from mosquito blood meals [15, 18–20], demonstrating its versatility for broad-scale pathogen surveillance across human, livestock, and wildlife interfaces. Molecular tools, particularly PCR coupled with high-resolution melting (HRM) analysis and ribosomal gene targets, such as 16S rRNA, have proven especially effective for this purpose, enabling sensitive and cost-efficient detection of genetically diverse pathogens directly from arthropod homogenates without the need for culture-based methods [6, 7, 21]. Insights derived from these vector–host–pathogen profiles are critical for guiding disease-control strategies [22] and position xenosurveillance as a powerful component of integrated One Health surveillance systems.

Although most research on mosquito-associated pathogens has focused on arboviruses and protozoa, such as *Plasmodium* spp., increasing attention is being directed toward the detection of tick-borne bacterial pathogens in mosquitoes as part of integrated biosurveillance efforts [23]. Recent molecular studies have identified *Rickettsia* DNA in blood-fed mosquitoes, suggesting that these insects may acquire tick-borne bacteria incidentally during blood feeding and could potentially act as mechanical carriers [24–26]. Specifically, *Anaplasma* spp. DNA has been detected in field-collected mosquitoes across multiple genera in Africa [27] and China [28], while *Candidatus* Neoehrlichia mikurensis has been identified across mosquito life stages, suggesting environmental or blood-meal acquisition [28]. Despite these findings from West and Central Africa and China, comparable data from East Africa remain entirely absent from the literature. While these detections do not imply biological transmission, they highlight the value of mosquitoes as passive sentinels for identifying areas of elevated pathogen circulation in livestock systems. In Kenya, *Anaplasma marginale*, *Anaplasma* spp., *Theileria* spp., and *Babesia* spp. have been molecularly confirmed in cattle and ticks collected from livestock and wildlife at wildlife–livestock interfaces [29–31], and specifically in cattle, ticks, and skin swabs at the Kajiado County study site [7], establishing that the pathogen community targeted in this study actively circulates among vertebrate hosts in these agro-pastoral landscapes. However, whether mosquitoes feeding on these hosts acquire and carry detectable pathogen DNA, and whether this can be exploited for sustainable xenosurveillance, has never been evaluated in the region. In this study, we collected mosquitoes around cattle enclosures at night to investigate their potential role in the epidemiology and surveillance of tick-borne pathogens. Specifically, we evaluated whether mosquito-derived blood meals can reveal the presence and distribution of Anaplasmataceae in livestock-dominated environments. To our knowledge, this represents the first study to apply xenosurveillance for tick-borne bacterial pathogens using mosquitoes in East Africa, addressing a clear gap in the regional surveillance literature and contributing evidence toward the integration of blood-feeding insects into One Health monitoring frameworks.

## Methods

### Study area and mosquito collection

**Fig. 1:**
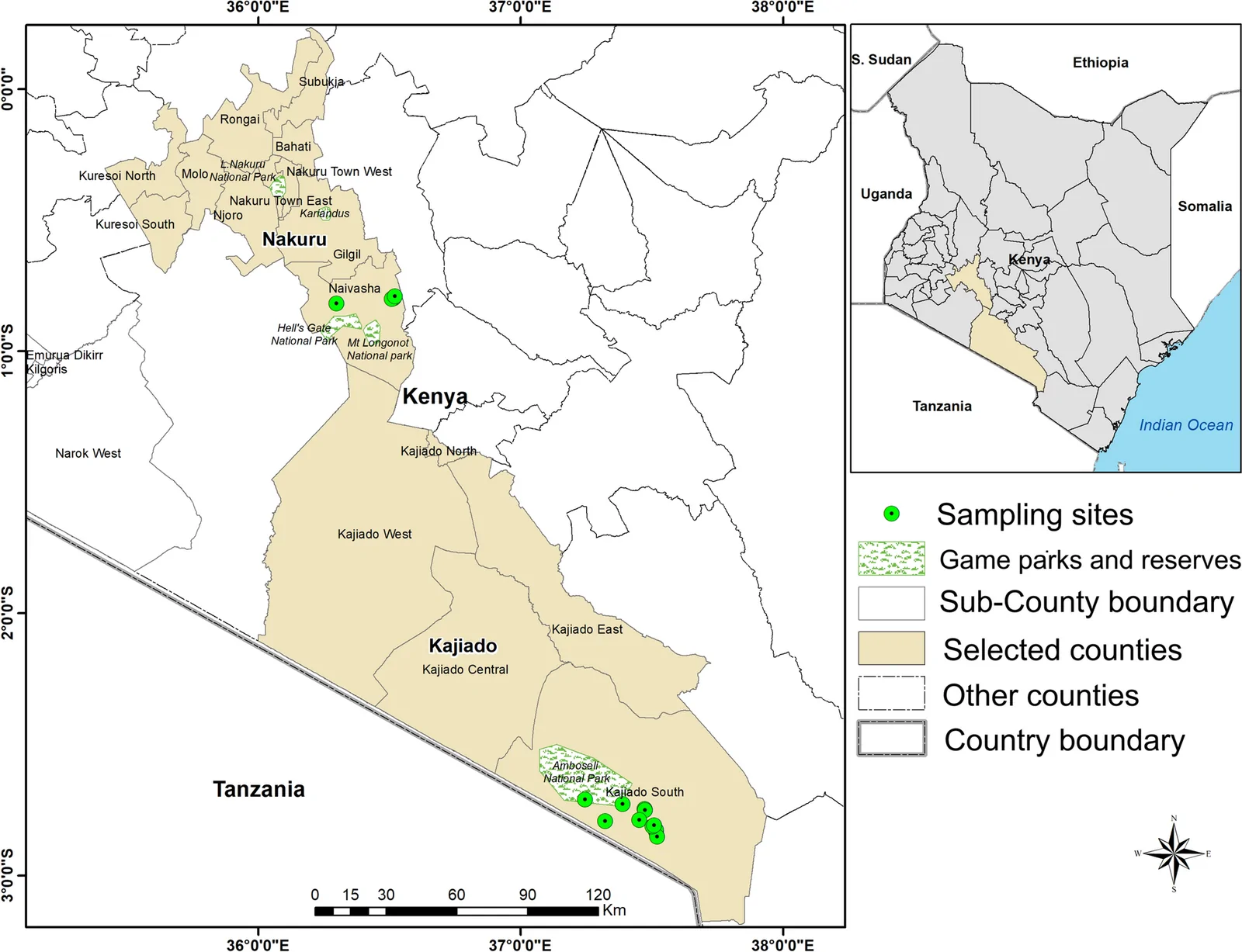
Map of the study area in the livestock–wildlife interface of Kajiado and Nakuru County, Kenya, showing mosquito sampling sites around cattle enclosures. The maps were created using the open-source software, QGIS v. 3.40.14

This cross-sectional study was conducted during the long rainy season (April 2024) in two livestock–wildlife interface regions of Kenya as part of a study by Getange et al. [7]: Amboseli in Kajiado County (approximately 2° 39′ S, 37° 15′ E; elevation ~ 1200 m above sea level) and Naivasha in Nakuru County (approximately 0° 43′ S, 36° 26′ E; elevation ~ 1880 m above sea level) (Fig. 1). Amboseli is an arid to semi-arid ecosystem with annual rainfall of 350–600 mm and mean temperatures of 18–28 °C, with open savannah, seasonal wetlands, and dispersed Maasai pastoralist settlements. It supports high densities of domestic livestock, primarily cattle (*Bos indicus*), goats (*Capra hircus*), and sheep (*Ovis aries*), alongside free-ranging wildlife, such as African buffalo (*Syncerus caffer*), elephants (*Loxodonta africana*), zebra (*Equus quagga*), giraffe (*Giraffa* spp.), and multiple antelope species. The close spatial overlap of these host communities facilitates the circulation of diverse vector-borne pathogens [32]. Naivasha, in contrast, is a sub-humid agro-ecological zone with higher mean annual rainfall (600–1200 mm), mean temperatures of 16–26 °C, and mixed livestock production, horticultural farming, and abundant wildlife associated with the Lake Naivasha ecosystem [33]. The proximity of diverse vertebrate host communities to permanent and seasonal water bodies at both sites creates favourable conditions for mosquito breeding and vector–host interactions.

Mosquitoes were collected around livestock enclosures overnight (6:00 pm–6:00 am) using Centres for Disease Control and Prevention (CDC) miniature light traps (Model 512, John Hock Co., Gainesville, Florida, USA) fitted with incandescent light bulbs and baited with carbonated dry ice (CO_2_) during the long rainy season in April 2024, a period associated with increased vector activity. Trapping was conducted in seven villages in Kajiado and six in Naivasha, with one village sampled per night. In each village, mosquito trapping was carried out in the same three homesteads, where cattle blood and tick samples were collected for the companion study by Getange et al [7]. Ten traps were deployed around livestock enclosures per homestead each night. CDC light traps were selected to maximise species diversity and overall capture rates within a standardised sampling framework; blood-fed individuals were subsequently identified and sorted for xenosurveillance analyses. While resting traps may yield higher proportions of blood-engorged mosquitoes, the CDC trap approach enabled concurrent assessment of mosquito community composition and pathogen screening. Traps were suspended approximately 1.5 m above ground level and positioned 5 m from cattle enclosures (locally known as bomas; traditional enclosures constructed from thorny bushes and wooden poles used to confine livestock overnight for protection from predators) to maximize the capture of host-seeking mosquitoes. Traps operated continuously overnight and were retrieved the following morning. Mosquito samples were linked to the collection sites by geo-coding using a GPS. Captured mosquitoes were temporarily immobilized using triethylamine and transported in liquid nitrogen to International Centre of Insect Physiology and Ecology (*icipe*) Martin Lüscher Emerging Infectious Diseases (ML-EID) Laboratory in Nairobi for identification and further molecular analysis.

### Mosquito morphological identification

Mosquitoes were identified morphologically to species level under a stereomicroscope using established taxonomic keys, including Edwards [34], Harbach [35], and Huang and Ward [36]. Following identification, specimens were pooled into groups of 1–25 individuals based on species, sex, collection site, and collection date. Blood-fed mosquitoes were processed individually and treated as pools of size 1, a deliberate decision consistent with the study’s primary objective of detecting and characterizing tick-borne bacterial pathogens from blood-meal-derived DNA. Mosquitoes of the same species were placed in sterile 1.5-mL microcentrifuge tubes (303 pools in total) and stored at − 80 °C until further molecular processing.

### DNA extraction of mosquito pools and individuals

Mosquito pools and individual mosquitoes were homogenized as whole specimens (without abdominal dissection) prior to DNA extraction to ensure thorough disruption of tissues. This whole-mosquito approach was adopted, because the primary objective was to detect pathogen DNA acquired through blood-feeding, consistent with established xenosurveillance protocols [15, 20]. Consequently, detected pathogen DNA may derive from the ingested blood-meal, residual host blood in the midgut, or, less likely, the mosquito’s own tissues. Specimens were not surface-sterilised prior to homogenization, as we did not aim to establish vector competence. Each pool was placed in a 1.5-mL microcentrifuge tube containing five 2.0-mm zirconia/yttria-stabilized beads (BioSpec, USA) and mechanically disrupted using a Mini Bead Beater 16 (BioSpec, Bartlesville, USA) for approximately 30 s at ~ 3450 rpm. Following homogenization, genomic DNA was extracted using the Isolate II Genomic DNA Kit (Bioline, UK) according to the manufacturer’s instructions. Extracted DNA from each pool was stored at − 20 °C until PCR screening.

### Mosquito blood-meal source identification

All blood-fed mosquito samples were individually analysed to determine the vertebrate host origin of the blood. Host identification was performed using PCR amplification of vertebrate mitochondrial markers, specifically cytochrome *b* (cyt *b*) and 16S rRNA (vert16S), which have been shown to reliably differentiate a wide range of vertebrate taxa [29]. For each specimen, 1 µL of extracted nucleic acid served as template in a 10 µL reaction mixture containing 2 µL of 5 × HOT FIREPol^®^ EvaGreen^®^ qPCR Mix (Solis BioDyne, Estonia) and 10 pmol of each primer. PCR amplification and high-resolution melting (HRM) analysis were conducted on a MIC PCR Cycler (BioMolecular Systems, Upper Coomera, Queensland, Australia) following previously optimized protocols [37]. DNA from known vertebrate hosts, including human, cattle, sheep, goat, elephant, and chicken, was included in each run as positive controls to support interpretation of melting profiles. Nuclease-free water was included as a non-template control in each assay to monitor for potential reagent contamination. Representative amplicons exhibiting distinct HRM profiles consistent with our positive controls were purified using an Exo-rSAP enzymatic cleanup (New England Biolabs, UK) and outsourced to Macrogen Europe (The Netherlands) for Sanger sequencing to confirm host identity.

### Screening of mosquito pools for *Anaplasma*, *Ehrlichia*, *Rickettsia*, *Theileria*, and *Babesia*

All mosquito DNA samples were screened for *Anaplasma*, *Ehrlichia*, *Rickettsia*, *Theileria*, and *Babesia* using PCR–high-resolution melting (HRM) analysis performed on a Mic PCR Cycler (BioMolecular Systems, Upper Coomera, Queensland, Australia). PCR–HRM was selected as the primary screening method, because it enables rapid, cost-effective differentiation of closely related pathogen species based on amplicon melting profiles and has been validated for tick-borne pathogen detection in previous studies in Kenya [6, 7, 21]. Genus-specific 16S rRNA primers for *Anaplasma* and *Ehrlichia*, and 18S rRNA primers for *Theileria* and *Babesia*, were used, because these ribosomal gene targets offer broad taxonomic coverage within their respective genera while providing sufficient sequence variability for species-level discrimination upon sequencing. Screening was conducted using previously published primer sets (Table 1), following the PCR–HRM protocol described by Mwamuye et al. [21] and Getange et al. [6, 7]. Primer pairs included those amplifying fragments of the 16S rRNA gene for *Anaplasma* spp. (AnaplasmaJVF/JVR) and *Ehrlichia* spp. (Ehrlichia16S F/R), and the *Rickettsia* spp. 16S rRNA gene (Rick16SF/R). In addition, the 18S rRNA gene of *Theileria* and *Babesia* spp. was amplified using RLB F/R primers. Each 10-µL HRM reaction consisted of 0.5 µL of each primer (10 pmol/µL), 2 µL of FIREPol^®^ EvaGreen^®^ HRM Master Mix (Solis BioDyne, Tartu, Estonia), 1 µL genomic DNA, and nuclease-free water to final volume. PCR cycling and HRM conditions followed previously described protocol [6, 21].

**Table 1:** Primers used for blood-meal analysis and molecular detection of tick-borne pathogens in mosquito pools

Primer name	Gene target	Target size (bp)	Sequence (5’–3’)	Reference
Cytb F Cytb R	Vertebrate cyt *b*	383	CCCCTCAGAATGATATTTGTCCTCA CATCCAACATCTCAGCATGATGAAA	[38]
Vert16S F Vert16S R	Vertebrate 16S	250	GAGAAGACCCTRTGGARCTT CGCTGTTATCCCTAGGGTA	[29]
Rick-F Rick-R	*Rickettsia* 16S rRNA	364	GAACGCTATCGGTATGCTTAACACA CATCACTCACTCGGTATTGCTGGA	[39]
120–2788 120–3599	*Rickettsia* ompB	856	AAACAATAATCAAGGTACTGT TACTTCCGGTTACAGCAAAGT	[40]
*Ehrlichia*16S F *Ehrlichia*16SR	*Ehrlichia* 16S rRNA	200	CGTAAAGGGCACGTAGGTGGACTA CACCTCAGTGTCAGTATCGAACCA	[41]
PER1 PER2	*Ehrlichia* 16S rRNA	451	TTTATCGCTATTAGATGAGCCTATG CTCTACACTAGGAATTCCGCTAT	[42]
EHR16SD 1492R	*Anaplasma*/*Ehrlichia* 16S rRNA	1030	GGTACCYACAGAAGAAGTCC GGTTACCTTGTTACGACTT	[43, 44]
*Anaplasma*JF *Anaplasma*JR	*Anaplasma* 16S rRNA	300	CGGTGGAGCATGTGGTTTAATTC CGRCGTTGCAACCTATTGTAGTC	[21]
RLB F RLB R	*Theileria*/*Babesia* 18S rRNA	460–520	GAGGTAGTGACAAGAAATAACAATA TCTTCGATCCCCTAACTTTC	[45]

All mosquito pools were screened regardless of blood-feeding status to provide a comprehensive assessment of pathogen detection across the collection. This approach allowed comparison of detection rates between blood-fed and non-blood-fed specimens and is consistent with established xenosurveillance study designs [15, 20]. The xenosurveillance interpretation, specifically the analysis of pathogen–host concordance, was restricted to individually processed blood-fed mosquitoes.

Samples with distinct HRM profiles were subjected to confirmatory PCR assays using EHR16SD–1492R primer pair for *Anaplasma* spp., and PER1–PER2 primers for *Ehrlichia* spp. (Table 1). These reactions were performed on a ProFlex PCR System (Applied Biosystems, Foster City, CA, USA) in a total volume of 15 µL, comprising 3 µL of 5 × HOT FIREPol^®^ Blend Master Mix (Solis BioDyne, Tartu, Estonia), 1.5 µL of template DNA, and 0.75 µL each of forward and reverse primers (10 pmol/µL). Thermocycling conditions followed previously published protocols [42–44]. Amplification success was assessed by electrophoresing 5 µL of PCR products on a 2% agarose gel pre-stained with ethidium bromide, followed by visualization under UV illumination. Remaining PCR products were purified using ExoSAP (New England Biolabs, UK) according to the manufacturer’s guidelines. Purified amplicons were then submitted to Macrogen Europe (The Netherlands) for Sanger sequencing.

### Phylogenetic and bioinformatic analyses

Forward and reverse sequence chromatograms obtained through Sanger sequencing were inspected, edited, and assembled into high-quality consensus sequences using Geneious Prime v2024.0.7 [46]. Consensus sequences were compared against publicly available reference sequences in the NCBI database (http://www.ncbi.nlm.nih.gov) through BLASTn searches [47] to verify taxonomic identities and assess similarity to known pathogen reference sequences. For evolutionary inference, multiple sequence alignments were generated using MAFFT, after which maximum-likelihood phylogenetic trees were reconstructed in PhyML v3.0 [48]. The optimal nucleotide substitution model for each dataset was selected automatically using the Akaike Information Criterion [49]. Resulting phylogenies were visualized and annotated in FigTree v1.4.4 [50]. All sequences generated in this study were deposited in the NCBI GenBank database under accession numbers PZ069361–PZ069368. Pathogen detection frequencies were expressed as the proportion of positive mosquito pools relative to the total number of pools screened for each pathogen group. In addition, minimum infection rates (MIR) were calculated as the number of positive pools divided by the total number of individual mosquitoes tested per species, multiplied by 1,000, to enable standardised comparison across species with different pool sizes.

## Results

### Morphological identification of mosquitoes

To contextualise pathogen detection patterns, we first describe the mosquito community composition across both study sites, as species composition directly influenced which pools were eligible for xenosurveillance analysis. A total of 4673 mosquitoes (303 pools) representing five genera (*Aedes*, *Anopheles*, *Culex*, *Mansonia,* and *Coquillettidia*), were collected in Amboseli (*n* = 546) and Naivasha (*n* = 4127) (Table 2). Overall, *Culex* was the dominant genus, accounting for 74.3% (*n* = 3473) of all collected mosquitoes, followed by *Mansonia* at 23.5% (*n* = 1100). In contrast, *Aedes* and *Anopheles* were comparatively less abundant, contributing 1.5% (*n* = 70) and 0.4% (*n* = 19) of the total catch, respectively, while *Coquillettidia aurites* represented 0.24% (*n* = 11). At the species level, the most abundant mosquitoes were *Culex pipiens* (*n* = 3220; 68.9%), followed by *Mansonia africana* (*n* = 976; 19.6%), and *Culex vansomereni* (*n* = 126; 2.7%). These three species collectively constituted over 92.5% of the entire mosquito collection. Naivasha collections were dominated by *Culex pipiens* (*n* = 2837; 68.7%), followed by *Mn. africana* (*n* = 942; 22.8%) and *Cx. vansomereni* (*n* = 126; 3.1%). In contrast, Amboseli was dominated by *Cx. pipiens* (*n *= 383; 70.1%), followed by *Cx. univittatus* (*n *= 90; 16.5%) and *Mn. africana* (*n *= 34; 6.2%).

**Table 2:** Summary of mosquito species trapped at the sampling sites in Kajiado and Naivasha counties of Kenya

			Amboseli		Naivasha		Total
Genus	Species	Pools	F	M	F	M	
*Aedes*	*Ae. aegypti*	1	2				2
	*Ae. hirsutus*	11	18		29		47
	*Ae. mcintoshi*	11	10		5		15
	*Ae. tarsalis*	2			3		3
	*Ae. tricholabis*	2			3		3
*Anopheles*	*An. coustani*	2			3		3
	*An. funestus*	3	1		2		3
	*An. gambiae*	1			1		1
	*An. pharoensis*	6			9	3	12
*Coquillettidia*	*Cq. aurites*	4			10	1	11
*Culex*	*Cx. annulirostris*	2			3		3
	*Cx. bitaeniorhynchus*	1			1		1
	*Cx. cinereus*	1	1				1
	*Cx. pipiens*	150	366	17	2827	10	3220
	*Cx. poicilipes*	3	1		3		4
	*Cx. tigripes*	1			1		1
	*Cx. univittatus*	16	90		15		105
	*Cx. vansomereni*	11			124	2	126
	*Cx. zombaensis*	2			12		12
*Mansonia*	*Mn. africana*	50	34		877	65	976
	*Mn. uniformis*	23	4	2	83	35	124
Total		303	527	19	4011	116	4673

### Mosquito blood-meal analysis

**Fig. 2:**
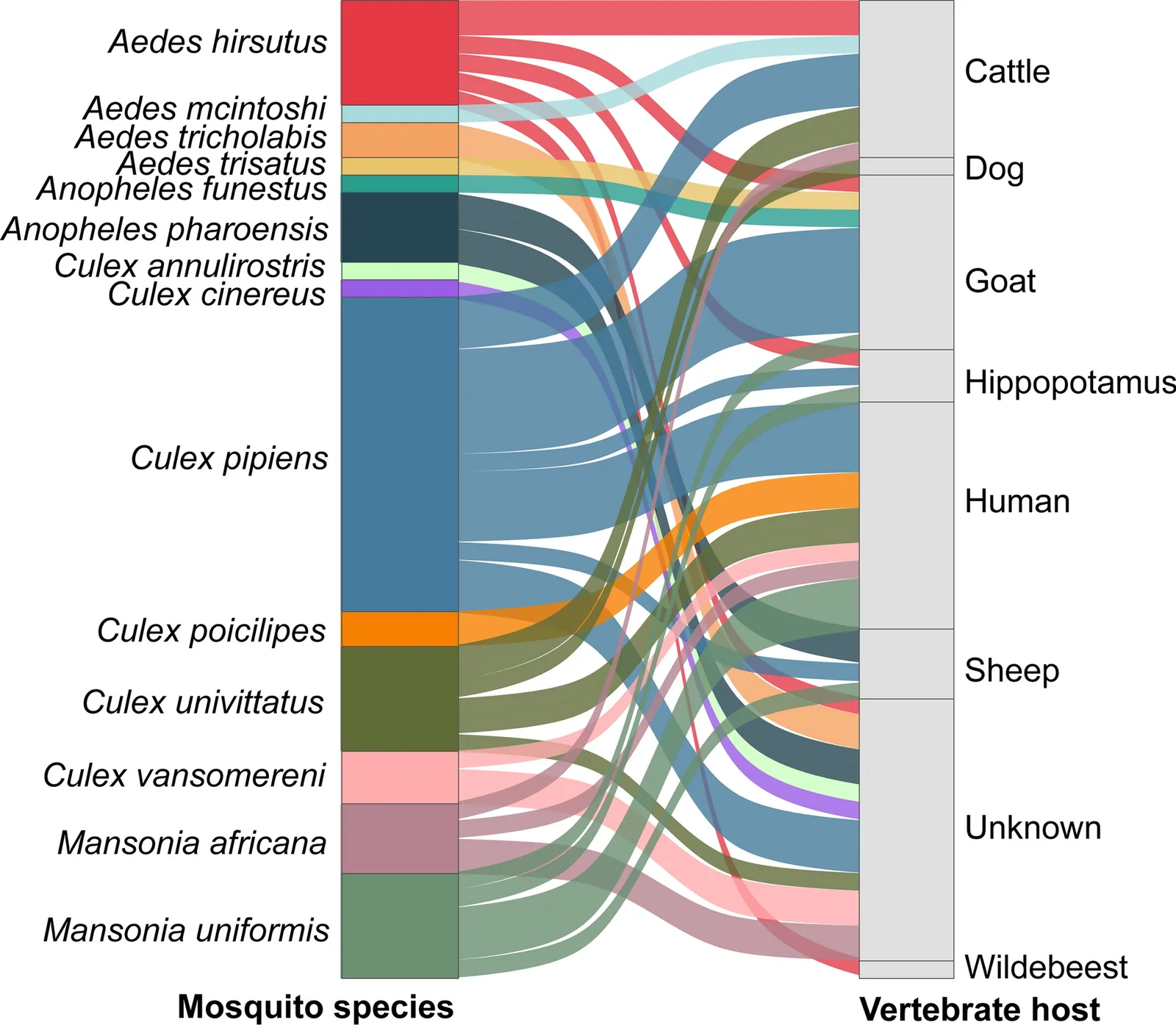
Alluvial diagram showing mosquito blood-feeding patterns and vertebrate host associations, visualized using the *bipartite* R package

Fifty-six mosquitoes that were visibly blood-fed representing eight species across four genera (*Culex, Aedes, Anopheles*, and *Mansonia*) were analysed for vertebrate blood-meal sources using cytochrome *b* sequencing. Vertebrate host DNA was successfully identified in 43 mosquitoes, representing a 76.8% success rate (*n* = 56). The detected vertebrate hosts included humans (*Homo sapiens*), cattle (*Bos taurus*), goats (*Capra hircus*), sheep (*Ovis aries*), hippopotamus (*Hippopotamus amphibius*), dog (*Canis lupus familiaris*), and wildebeest (*Connochaetes taurinus*) (Fig. 2), while the remaining 13 samples failed to amplify with any of the vertebrate primers used, likely due to advanced blood-meal digestion, low DNA quantity, or host species falling outside the primer amplification range. Overall, human (23.2%, *n* = 13) was the most frequently detected vertebrate blood-meal source, followed closely by cattle (19.6%, *n *= 11), goats (17.9%, *n* = 10), and sheep (7.1%, *n* = 4). Wildlife-derived blood meals were less common and included *Hippopotamus* (5.4%, *n *= 3) and wildebeest (1.9%,* n *= 1), while dog blood was detected in a single sample (1.9%, *n *= 1). *Culex* species, particularly *Cx. pipiens* and *Cx. univittatus*, showed the greatest diversity of host use and accounted for most livestock- and human-derived blood meals, whereas *Mansonia* and *Aedes* species contributed mainly to wildlife- and mixed-interface feeding patterns. In Nakuru, goats were the primary host (*n* = 9), while in Kajiado, cattle were the most frequent source of blood meals (*n* = 9) (Table S1).

### Pathogen detection in mosquito pools

**Fig. 3:**
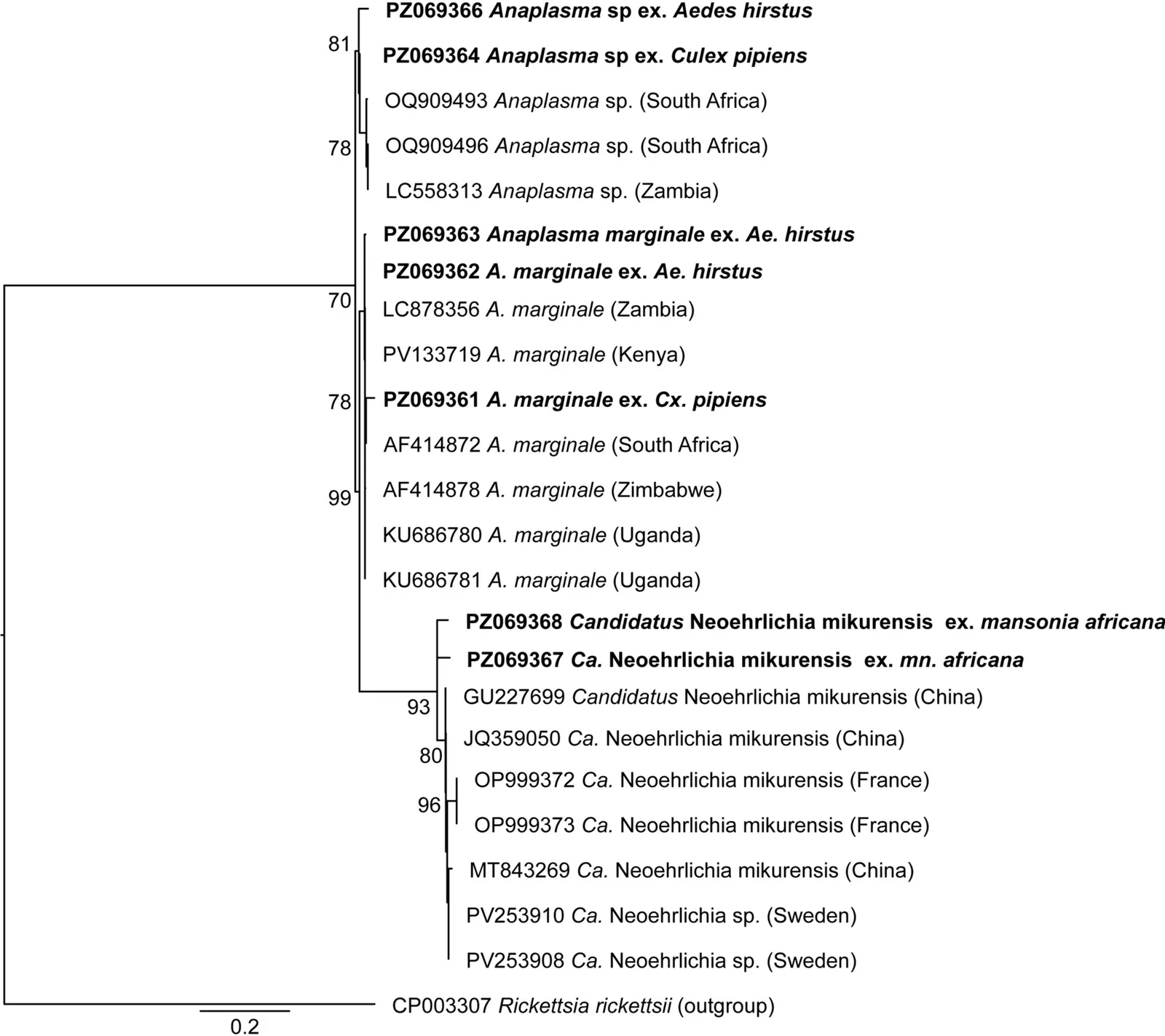
Maximum-likelihood phylogenetic tree of *Anaplasma* and *Candidatus* Neoehrlichia mikurensis 16S rRNA gene (1030 bp) gene sequences (some identical sequences are represented by a single entry). GenBank accession numbers, species identifications, and country of origin in brackets. Sequences from this study are bolded. Bootstrap values at the major nodes are of percentage agreement among 1000 replicates

Of the 303 pools screened, all eight pools yielding positive pathogen detections were blood-fed individuals processed as single-mosquito pools; no tick-borne pathogens were detected in any non-blood-fed pool, irrespective of species or site. Screening of mosquito pools for tick-borne pathogens revealed low but notable detection of *Anaplasma* and *Neoehrlichia* spp. in mosquito species from Kajiado County, and none from Naivasha County. *Culex pipiens* had the highest diversity of detections, with *Anaplasma marginale* identified in 1/150 pools (0.7%; MIR = 0.31) and an unclassified *Anaplasma* sp. (2/150; 1.4%; MIR = 0.62). In contrast, *Aedes hirsutus* showed comparatively higher pool positivity rates, with *A. marginale* (2/11; 18.2%; MIR = 42.55) and *Anaplasma* sp. (1/11; 9.1%; MIR = 21.28). Phylogenetic analysis based on partial 16S rRNA gene sequences showed that *A. marginale* sequences generated in this study clustered closely with reference sequences from Kenya (PV133719; 99.89%) and Uganda (KU686780; 99.79%). Similarly, *Anaplasma* sp. sequences aligned with reference sequences from South Africa (OQ909493; 99.44%) and Zambia (LC558313; 99.55%) (Fig. 3).

Notably, *Mn. africana* was exclusively positive for *Candidatus* Neoehrlichia mikurensis (2/50; 4%), marking the only occurrence of this emerging pathogen within the mosquito collection (Tables 3and 4). Sequences identified as *Ca.* Neoehrlichia mikurensis clustered with reference sequences from China (GU227699; 98.1% and JQ359050; 98.1%), supporting their taxonomic assignment (Fig. 3). We did not detect any *Rickettsia*, *Theileria*, or *Babesia* DNA in this study.

**Table 3:** Pooled positivity rates and minimum infection rates (MIR) of tick-borne pathogens detected across all screened mosquito pools (including non-blood-fed pools and blood-fed individuals processed as pools of one) from Kajiado County, Kenya

Pathogen detected (No. of pools)	Positive pools	Pool positivity (%)	95% CI (lower–upper)	MIR (per 1000)
*Culex pipiens* (*n* = 150)				
*Anaplasma marginale*	1	0.67%	0.017% – 3.66%	0.31
*Anaplasma* sp.	2	1.33%	0.16% – 4.73%	0.62
*Aedes hirsutus* (*n* = 11)				
*Anaplasma marginale*	2	18.18%	2.28% – 51.78%	42.55
*Anaplasma* sp.	1	9.09%	0.23% – 41.28%	21.28
*Mansonia africana* (*n* = 50)				
*Candidatus* Neoehrlichia mikurensis	2	4.00%	0.49% – 13.71%	2.05

**Table 4:** Detailed description of individual tick-borne pathogen-positive mosquito samples from Kajiado County, Kenya, including mosquito species, blood-meal source, pathogen identity, sequence similarity, and GenBank accession numbers

Sample ID	Mosquito species	Blood-meal source	Pathogen	Identity (%)	Sequence length (bp)	GenBank accession
MSQ278	*Culex pipiens*	Unknown	*Anaplasma marginale*	99.7	660	PZ069361
MSQ137	*Aedes hirsutus*	Cattle	*Anaplasma* marginale	99.9	952	PZ069362
MSQ297	*Aedes hirsutus*	Cattle	*Anaplasma* marginale	99.8	947	PZ069363
MSQ120	*Culex pipiens*	Goat	*Anaplasma* sp.	99.5	917	PZ069364
MSQ163	*Culex pipiens*	Goat	*Anaplasma* sp.	99.4	910	PZ069365
MSQ273	*Aedes hirsutus*	Goat	*Anaplasma* sp.	100	391	PZ069366
MSQ201	*Mansonia africana*	Cattle	*Candidatus* Neoehrlichia mikurensis	98.1	377	PZ069367
MSQ207	*Mansonia africana*	Unknown	*Ca.* Neoehrlichia mikurensis	98.1	372	PZ069368

Tick-borne pathogen-positive mosquito pools were identified in three mosquito species, and their associated blood-meal sources were determined by cytochrome *b* analysis (Tables 4 and S1). *Anaplasma marginale* and *Anaplasma* sp. were detected in *Cx. pipiens* and *Ae. hirsutus*, whereas *Ca.* Neoehrlichia mikurensis was detected in *Mn. africana*. In *Cx. pipiens*, *Anaplasma* sp. was identified in two pools that had fed on goats, and *A. marginale* was detected in one pool with an undetermined blood-meal source, with sequence identities of 99.7–99.9% and amplicon lengths ranging from 660 to 952 bp. In *Ae. hirsutus*, *A. marginale* was detected in two pools that had fed on cattle, while *Anaplasma* sp. was detected in one pool with a goat-derived blood-meal, with sequence identities of 99.4–100% and fragment lengths of 391–917 bp. *Candidatus* Neoehrlichia mikurensis was detected in two *Mn. africana* pools, one associated with a cattle blood-meal and one with an unidentified host, showing 98.1% sequence identity over 372–377 bp. Overall, the detected tick-borne pathogens were associated primarily with livestock-derived blood meals, particularly cattle and goats, with sequence identity values ≥ 98.1%, providing the primary line of evidence that detected DNA derived from the ingested blood-meal rather than from environmental contamination or the mosquito's own tissues.

## Discussion

Here, we investigated the feasibility of using blood-feeding mosquitoes, for the surveillance of pathogens in livestock at a human–wildlife interface. We detected *A. marginale*, an unclassified *Anaplasma* sp., and to our knowledge for the first time, *Ca.* Neoehrlichia mikurensis in mosquito pools collected around cattle enclosures in Kajiado County, one of the two counties sampled in this study. The findings provide evidence that mosquitoes in livestock-dense ecosystems may acquire and indirectly reflect the circulation of bacterial pathogens traditionally associated with tick vectors. Although mosquitoes are not recognized as biological vectors of these bacteria, our results are consistent with previous field studies demonstrating the presence of tick-borne bacterial DNA in field-collected mosquitoes [25, 28]. Collectively, these observations support the concept that mosquitoes can function as ecological sentinels of vertebrate infections in pastoral landscapes. Our findings add to a growing body of literature indicating that blood-feeding arthropods, including mosquitoes and other hematophagous insects, may serve as valuable, non-invasive tools for monitoring pathogen circulation in animal populations [51, 52].

Interestingly, all pathogen detections in this study originated exclusively from Kajiado County, despite Naivasha contributing 88% of the total mosquito collection (4127 of 4673 specimens). Possible explanations include mosquito species composition differed between sites, with livestock-feeding species potentially more abundant in Kajiado's pastoral landscape, increasing the probability of blood-meal acquisition from tick-infested hosts. Given that tick-borne pathogen DNA in mosquitoes represents blood-meal content rather than vector infection, variation in livestock management practices that may influence tick burdens and pathogen exposure may also contribute [53]. In addition, the distinct agro-ecological characteristics of Naivasha, a sub-humid zone with mixed farming [33], versus the arid pastoral landscape of Amboseli [32] may influence vector–host–pathogen dynamics. Moreover, sampling was restricted to a single month (April 2024) during the long rains, when mosquito abundance and host-seeking activity are typically high. Mosquito community composition and abundance fluctuate seasonally in East African highlands and semi-arid systems [54, 55], and the relative activity of different species, particularly those that contributed pathogen detections in this study (*Ae. hirsutus*, *Mn. africana),* may differ between wet and dry seasons. Indeed, recent work in the Amboseli ecosystem of Kajiado County demonstrated that mosquito abundance peaked in April 2024, with land use and seasonal weather factors both significantly shaping species composition across the landscape [54]. Mosquito species distribution also varies markedly across Kenya's ecological zones, with semi-arid areas supporting distinct fauna compared to sub-humid zones [55]. This could have influenced both the mosquito communities sampled and the pathogens detected at each site. Future studies with balanced sampling designs across sites would help clarify whether this pattern reflects genuine spatial variation in pathogen circulation.

This study detected *A. marginale* in *Cx. pipiens* that fed on unknown host and *Ae. hirsutus* that fed on cattle, suggesting exposure of livestock to this pathogen, and that mosquito blood meals can reflect host infection patterns. Circulation of *A. marginale* in the region was further supported by parallel findings from a complementary study that molecularly confirmed presence of the pathogen in cattle blood samples collected from same cattle enclosures as mosquitoes in this study using direct host-based diagnostics [7], though the infection rates were higher in blood (24.6%) than mosquitoes (1.9%). All *A. marginale* positive mosquitoes were detected in enclosures that contained *A. marginale*-positive cattle. The agreement between pathogen detection in mosquito blood meals and cattle blood [7] provides clear empirical evidence that mosquito-based xenosurveillance can reliably reflect livestock infection status. This finding is noteworthy given the pathogen’s well-established role in bovine anaplasmosis and its economic impact across sub-Saharan Africa [56]. Importantly, while previous studies have shown that various hematophagous insects may transiently harbour *Anaplasma* DNA following blood feeding [27], and that *Anaplasma* and *Rickettsia* species can be maintained in mosquito cell lines under laboratory conditions [57, 58], these observations do not imply biological transmission by mosquitoes in nature. Our detections are interpreted solely as reflecting recent blood feeding on infected vertebrate hosts, not as evidence of vector competence or participation in transmission cycles. Similarly, the discovery of unclassified *Anaplasma* sp. in a mosquito that fed on goat mirrors previous findings of un-characterized *Anaplasma* diversity in livestock in Kenya [7, 59], expanding the scope of known circulation and suggesting broad ecological reservoirs. Host–pathogen associations observed in this study were highly consistent: *A. marginale* detections in *Ae. hirsutus* were from cattle-fed individuals, while *Anaplasma* sp. was exclusively associated with goat-derived blood meals in both *Cx. pipiens* and *Ae. hirsutus*. This concordance between pathogen identity and expected vertebrate host strengthens the biological plausibility of the xenosurveillance detections and suggests that mosquito blood meals accurately capture host-specific infection patterns.

We detected *Ca.* Neoehrlichia mikurensis, a bacterium first described as a human pathogen in Sweden in 2010 [60], exclusively in *Mn. africana* that fed on cattle and unknown host, representing the first report of this pathogen in mosquitoes in Africa. While *Ca.* Neoehrlichia mikurensis is traditionally considered a tick-borne pathogen transmitted primarily by *Ixodes* species in Europe and Asia, detection in mosquitoes has been previously reported. Guo et al. [28] identified *Ca.* Neoehrlichia mikurensis DNA in multiple mosquito species across all life stages, eggs, larvae, pupae, and adults, in China, suggesting that mosquitoes may acquire this bacterium through environmental exposure or blood meals from infected vertebrate hosts. Previous studies have documented *Ca.* Neoehrlichia mikurensis in rodents [61, 62], bovine blood [63], ticks in Africa [64] and Europe [65], and humans across multiple European countries [60, 66–71]. Given that *Mn. africana* readily feeds on both livestock and humans in peri-agricultural settings, our detection suggests that mosquitoes may serve as environmental sentinels, providing a non-invasive reflection of local pathogen circulation. Our blood-meal data show that *Mn. africana* at the Kajiado site fed on both livestock and humans, an observation of epidemiological relevance given the zoonotic status of *Ca. Neoehrlichia mikurensis*. However, this reflects feeding behaviour rather than evidence of transmission. Nonetheless, these findings highlight the One Health value of xenosurveillance in identifying settings, where zoonotic pathogen circulation and cross-interface feeding coincide, supporting targeted serological screening of both livestock and human populations in the area. However, important knowledge gaps remain. Capacity of *Ca.* Neoehrlichia mikurensis to survive or replicate within mosquito tissues has not been assessed, and the potential for mechanical transmission has not been evaluated. In addition, the contribution of wildlife or rodent reservoirs in Kenyan agro-pastoral landscapes remains unknown. Future research should, therefore, focus on (i) tracing *Ca.* Neoehrlichia mikurensis in livestock, rodents, and ticks around sampling sites, (ii) experimentally testing bacterial viability and persistence in mosquito tissues, including salivary glands, and (iii) determining whether mosquito-derived detections reflect genuine exposure or incidental carriage.

However, we did not detect *Rickettsia*, *Theileria*, or *Babesia* DNA in any mosquito pool, despite screening all 303 pools with validated primer sets. The absence of *Rickettsia* is consistent with the low prevalence of rickettsial agents reported in livestock in the study region. The failure to detect *Theileria* and *Babesia*, despite their documented prevalence in cattle in the same study area [7], may reflect inherent limitations of xenosurveillance for intracellular parasites that sequester in erythrocytes and maintain relatively low circulating DNA loads in peripheral blood. This observation suggests that mosquito-based xenosurveillance may be better suited for detecting bacterial pathogens with higher bacteraemia levels than for apicomplexan haemoparasites and highlights the need to consider pathogen biology when evaluating the sensitivity of this approach.

Our findings support the growing recognition of mosquitoes as “flying syringes” or biological samplers capable of capturing vertebrate pathogen profiles without implying vector competence. Previous studies have reported presence of pathogen nucleic acid in mosquito midgut days after feeding [15, 72], highlighting the possibility of exploiting this retention period for molecular detection of pathogens. Our findings are consistent with xenosurveillance approaches using other hematophagous insects that have revealed cryptic infection reservoirs across wildlife and livestock populations [9, 11, 15, 20, 73, 74]. Therefore, our data reflect environmental circulation rather than biological transmission, demonstrating that mosquitoes can function as scalable, cost-efficient biosamplers, particularly where direct host sampling is difficult, ethically constrained, or expensive. Future laboratory studies should quantify factors affecting detection, such as the decay kinetics of pathogen nucleic acids in the mosquito gut, the time interval between feeding and sample processing, and the pathogen load in the host at the time of feeding, to refine detection probabilities and optimize sampling designs.

However, several limitations should be considered when interpreting these findings. Detection of bacterial DNA in mosquitoes reflects recent blood feeding on infected hosts and does not indicate pathogen viability, replication, or transmission potential. Therefore, results from this study should be interpreted strictly within a xenosurveillance framework. The use of pooled samples and variability in the interval between blood feeding, mosquito capture, and processing may have influenced detection sensitivity due to dilution effects or nucleic acid degradation. In addition, because mosquitoes were collected near cattle enclosures and specimens were not surface-sterilised prior to homogenization, the possibility that external contamination with environmental pathogen DNA cannot be entirely excluded. Whole mosquitoes were processed without abdominal dissection; hence we cannot definitively attribute detected pathogen DNA solely to the blood-meal. However, the obligate intracellular nature of Anaplasmataceae makes it unlikely that these bacteria originated from the mosquito’s external surface or endogenous microbiome. Moreover, the strong concordance between pathogen identity and expected vertebrate host in individually processed blood-fed specimens (e.g., *A. marginale* in cattle-fed mosquitoes, *Anaplasma* sp. in goat-fed mosquitoes) argues strongly for a blood-meal origin, as incidental carriage would not be expected to correlate with host identity. Furthermore, pathogen detection rates for some species, particularly *Ae. hirsutus* (*n* = 11 pools), were based on small sample sizes and should not be directly compared with rates from more extensively sampled species. Sampling was limited to a single night per village, which may not fully capture nightly variation in mosquito abundance and host-seeking activity, and could have influenced species composition and pathogen detection rates at individual sites. Finally, the cross-sectional design precludes assessment of temporal dynamics in pathogen circulation. These figures should be interpreted as preliminary observations requiring confirmation in larger collections. Future studies incorporating individual mosquito analyses, longitudinal sampling, and controlled laboratory experiments will be important for refining interpretation and strengthening mosquito-based xenosurveillance approaches.

## Conclusions

Our results provide preliminary evidence that mosquito-based xenosurveillance can detect tick-borne bacterial pathogens circulating in livestock at human–wildlife interfaces in Kenya. The strong concordance between pathogen identity and vertebrate host species in blood-fed mosquitoes supports the biological plausibility of this approach and demonstrates that mosquito-derived blood-meal DNA can serve as a reliable proxy for pathogen presence in vertebrate hosts. Notably, the detection of *Ca.* Neoehrlichia mikurensis, a known zoonotic pathogen, in mosquitoes feeding across the livestock–human interface highlights the broader One Health relevance of this method, pointing to areas, where human exposure risk may deserve closer attention. Mosquito-derived sampling has clear potential to complement existing surveillance tools, particularly in agro-pastoral systems, where direct host sampling is logistically difficult or economically prohibitive. Future studies should employ balanced multi-site sampling designs, longitudinal monitoring across seasons, and whole-genome or multi-locus sequencing approaches to refine the sensitivity, specificity, and operational scalability of mosquito-based xenosurveillance across Kenya's diverse ecological zones.

## Data Availability

The sequences generated and analysed during the current study have been deposited at the NCBI GenBank database under accession PZ069361–PZ069368.

## References

[1] Vieira CJSP, Gyawali N, Onn MB, Shivas MA, Shearman D, Darbro JM, et al. Mosquito bloodmeals can be used to determine vertebrate diversity, host preference, and pathogen exposure in humans and wildlife. Sci Rep. 2024;14:23203. 10.1038/s41598-024-73820-y. 39369026 PMC11455984

[2] Daszak P, Cunningham AA, Hyatt AD. Emerging infectious diseases of wildlife-threats to biodiversity and human health. Science. 2000;287:443–9. 10.1126/science.287.5452.443. 10642539

[3] Clements BW, Casani JAP. Emerging and reemerging infectious disease threats. In: Disasters and public health. 1st ed. Elsevier; 2016;4:245–265. 10.1016/B978-0-12-801980-1.00010-6.

[4] Jones KE, Patel NG, Levy MA, Storeygard A, Balk D, Gittleman JL, et al. Global trends in emerging infectious diseases. Nature. 2008;451:990–3. 10.1038/nature06536. 18288193 PMC5960580

[5] Cutler SJ, Fooks AR, van der Poel WH. Public health threat of new, reemerging, and neglected zoonoses in the industrialized world. Emerg Infect Dis. 2010;16:1–7. 10.3201/eid1601.081467. 20031035 PMC2874344

[6] Getange D, Bargul JL, Kanduma E, Collins M, Bodha B, Denge D, et al. Ticks and tick-borne pathogens associated with dromedary camels (*Camelus dromedarius*) in northern Kenya. Microorganisms. 2021;9:1414. 10.3390/microorganisms9071414. 34209060 PMC8306667

[7] Getange D, Mukaratirwa S, Bargul JL, Khogali R, Ng’iela J, Kabii J, et al. Molecular characterisation of tick-borne pathogens in cattle in Kenya: insights from blood, ticks, and skin swab analyses. BMC Vet Res. 2025;21:552. 10.1186/s12917-025-05014-1. 41039537 PMC12492888

[8] Makwarela TG, Seoraj-Pillai N, Nangammbi TC. Distribution and prevalence of ticks and tick-borne pathogens at the wildlife-livestock interface in Africa: a systematic review. Vet Sci. 2025;12:364. 10.3390/vetsci12040364. 40284866 PMC12031468

[9] De Marco MF, Brugman VA, Hernández-Triana LM, Thorne L, Phipps LP, Nikolova NI, et al. Detection of *Theileria orientalis* in mosquito blood meals in the United Kingdom. Veterinary Parasitol. 2016;15:31–6. 10.1016/j.vetpar.2016.09.012 27809975

[10] Kidambasi KO, Masiga DK, Villinger J, Carrington M, Bargul JL. Detection of blood pathogens in camels and their associated ectoparasitic camel biting keds, *Hippobosca camelina*: the potential application of keds in xenodiagnosis of camel haemopathogens. AAS Open Research. 2020;20:164. 10.12688/aasopenres.13021.1 PMC7243205 32510036

[11] Muita JW, Bargul JL, Makwatta JO, Ngatia EM, Tawich SK, Masiga DK, et al. *Stomoxys* flies (Diptera, Muscidae) are competent vectors of *Trypanosoma evansi*, *Trypanosoma vivax*, and other livestock hemopathogens. PLoS Pathog. 2025;21:e1012570. 10.1371/journal.ppat.1012570. 40327665 PMC12080930

[12] Bargul JL, Kidambasi KO, Getahun MN, Villinger J, Copeland RS, Muema JM, et al. Transmission of 'Candidatus Anaplasma camelii’ to mice and rabbits by camel-specific keds. Hippobosca camelina PLoS Negl Trop Dis. 2021;15:e0009671. 10.1371/journal.pntd.0009671. 34398891 PMC8389426

[13] Schaffner F, Medlock JM, Van Bortel W. Public health significance of invasive mosquitoes in Europe. Clin Microbiol Infect. 2013;19:685–92. 10.1111/1469-0691.12189. 23574618

[14] Lehmann T, Kouam C, Woo J, Diallo M, Wilkerson R, Linton YM. The African mosquito-borne diseasosome: geographical patterns, range expansion and future disease emergence. Proc Biol Sci. 2023;290:20231581. 10.1098/rspb.2023.1581. 38018102 PMC10685135

[15] Grubaugh ND, Sharma S, Krajacich BJ, Fakoli LS III, Bolay FK, Diclaro JW II, et al. Xenosurveillance: a novel mosquito-based approach for examining the human-pathogen landscape. PLoS Negl Trop Dis. 2015;9:e0003628. 10.1371/journal.pntd.0003628. 25775236 PMC4361501

[16] Kent RJ. Molecular methods for arthropod bloodmeal identification and applications to ecological and vector-borne disease studies. Mol Ecol Resour. 2009;9:4–18. 10.1111/j.1755-0998.2008.02469.x. 21564560

[17] Wisely SM, Torhorst CW, Botero-Cañola S, Atsma H, Burkett-Cadena ND, Reeves LE. Evaluation of mosquito blood meals as a tool for wildlife pathogen surveillance. Pathogens. 2025;14:792. 10.3390/pathogens14080792. 40872302 PMC12389132

[18] Barbazan P, Thitithanyanont A, Missé D, Dubot A, Bosc P, Luangsri N, et al. Detection of H5N1 avian influenza virus from mosquitoes collected in an infected poultry farm in Thailand. Vector Borne Zoonotic Dis. 2008;8:105–9. 10.1089/vbz.2007.0142. 18279078

[19] Ng TF, Willner DL, Lim YW, Schmieder R, Chau B, Nilsson C, et al. Broad surveys of DNA viral diversity obtained through viral metagenomics of mosquitoes. PLoS ONE. 2011;6:e20579. 10.1371/journal.pone.0020579. 21674005 PMC3108952

[20] Fauver JR, Weger-Lucarelli J, Fakoli LS 3rd, Bolay K, Bolay FK, Diclaro JW 2nd, et al. Xenosurveillance reflects traditional sampling techniques for the identification of human pathogens: a comparative study in West Africa. PLoS Negl Trop Dis. 2018;12:e0006348. 10.1371/journal.pntd.0006348. 29561834 PMC5880402

[21] Mwamuye MM, Kariuki E, Omondi D, Kabii J, Odongo D, Masiga D, et al. Novel *Rickettsia* and emergent tick-borne pathogens: a molecular survey of ticks and tick-borne pathogens in Shimba Hills National Reserve. Kenya Ticks Tick Borne Dis. 2017;8:208–18. 10.1016/j.ttbdis.2016.09.002. 28011185

[22] Chaves LF, Harrington LC, Keogh CL, Nguyen AM, Kitron UD. Blood feeding patterns of mosquitoes: random or structured? Front Zool. 2010;7:3. 10.1186/1742-9994-7-3. 20205866 PMC2826349

[23] Meireles ACA, Rios FGF, Feitoza LHM, da Silva LR, Julião GR. Non-destructive methods of pathogen detection: importance of mosquito integrity in studies of disease transmission and control. Pathogens. 2023;12:816. 10.3390/pathogens12060816. 37375506 PMC10302199

[24] Socolovschi C, Pages F, Ndiath MO, Ratmanov P, Raoult D. *Rickettsia* species in African Anopheles mosquitoes. PLoS ONE. 2012;7:e48254. 10.1371/journal.pone.0048254. 23118963 PMC3484133

[25] Zhang J, Lu G, Li J, Kelly P, Li M, Wang J, et al. Molecular detection of *Rickettsia felis* and *Rickettsia bellii* in mosquitoes. Vector Borne Zoonotic Dis. 2019;19:802–9. 10.1089/vbz.2019.2456. 31306085

[26] Maina AN, Klein TA, Kim HC, Chong ST, Yang YU, Mullins K, et al. Molecular characterization of novel mosquito-borne *Rickettsia *spp. from mosquitoes collected at the Demilitarized Zone of the Republic of Korea. PLoS one. 2017;12:e0188327. 10.1371/journal.pone.0188327 PMC5695765 29155880

[27] Lindh JM, Terenius O, Faye I. 16S rRNA gene-based identification of midgut bacteria from field-caught *Anopheles gambiae* sensu lato and* A. funestus* mosquitoes reveals new species related to known insect symbionts. Applied and environmental microbiology. 2005;71:7217–23. 10.1128/AEM.71.11.7217-7223.2005 PMC1287614 16269761

[28] Guo WP, Tian JH, Lin XD, Ni XB, Chen XP, Liao Y, et al. Extensive genetic diversity of Rickettsiales bacteria in multiple mosquito species. Sci Rep. 2016;6:38770. 10.1038/srep38770. 27934910 PMC5146937

[29] Omondi D, Masiga DK, Ajamma YU, Fielding BC, Njoroge L, Villinger J. Unravelling host–vector–arbovirus interactions by two-gene high-resolution melting mosquito blood meal analysis in a Kenyan wildlife–livestock interface. PLoS ONE. 2015;10:e0134375. 10.1371/journal.pone.0134375. 26230507 PMC4521840

[30] Adjou Moumouni PF, Aboge GO, Terkawi MA, Masatani T, Cao S, Kamyingkird K, et al. Molecular detection and characterization of *Babesia bovis, Babesia bigemina, Theileria* species and *Anaplasma marginale* isolated from cattle in Kenya. Parasit Vectors. 2015;8:496. 10.1186/s13071-015-1106-9. 26420543 PMC4589125

[31] Okal MN, Odhiambo BK, Otieno P, Bargul JL, Masiga D, Villinger J, et al. *Anaplasma* and *Theileria* pathogens in cattle of Lambwe Valley, Kenya: a case for pro-active surveillance in the wildlife-livestock interface. Microorganisms. 2020;8:1830. 10.3390/microorganisms8111830. 33233713 PMC7699859

[32] Chebor KO, Ichani FX. An Assessment of National and County Government Strategic Partnership in the Implementation of Paris Agreement on Climate Change: A Case of Kajiado County, Kenya (2013–2023). J African Interdisciplin Stud. 2025;9:233–46.

[33] Mbugua JK. Urban greening and nature based solutions potential in mitigating climate change impacts in municipalities. J Cities Infrastru. 2025;1:1–8.

[34] Edwards FW. Mosquitoes of the Ethiopian Region. III.-Culicine adults and pupae 1941.

[35] Harbach RE. The Culicidae (Diptera): a review of taxonomy, classification and phylogeny. Contrib Am Entomol Inst. 1988;24:1–240.

[36] Huang YM, Ward RH. A pictorial key for the identification of the mosquitoes associated with yellow fever in Africa. Mosq Syst. 1981;13:138–49.

[37] Ouso DD, Otiende MY, Jeneby MM, Oundo JW, Bargul JL, Miller SE, et al. Three-gene PCR and high-resolution melting analysis for differentiating vertebrate species mitochondrial DNA for biodiversity research and complementing forensic surveillance. Sci Rep. 2020;10:4741. 10.1038/s41598-020-61600-3. 32179808 PMC7075967

[38] Boakye DA, Tang J, Truc P, Merriweather A, Unnasch TR. Identification of blood meals in haematophagous Diptera by cytochrome B heteroduplex analysis. Med Vet Entomol. 1999;13:282–7. 10.1046/j.1365-2915.1999.00193.x. 10514054

[39] Nijhof AM, Bodaan C, Postigo M, Nieuwenhuijs H, Opsteegh M, Franssen L, et al. Ticks and associated pathogens collected from domestic animals in the Netherlands. Vector Borne Zoonotic Dis. 2007;7:585–95. 10.1089/vbz.2007.0130. 17979540

[40] Roux V, Raoult D. Phylogenetic analysis of members of the genus *Rickettsia* using the gene encoding the outer-membrane protein rOmpB (ompB). Int J Syst Evol Microbiol. 2000;50:1449–55. 10.1099/00207713-50-4-1449. 10939649

[41] Tokarz R, Kapoor V, Samuel JE, Bouyer DH, Briese T, Lipkin WI. Detection of tick-borne pathogens by MassTag polymerase chain reaction. Vector Borne Zoonotic Dis. 2009;9:147–51. 10.1089/vbz.2008.0088. 18800864 PMC2976645

[42] Goodman JL, Nelson C, Vitale B, Madigan JE, Dumler JS, Kurtti TJ, et al. Direct cultivation of the causative agent of human granulocytic ehrlichiosis. N Engl J Med. 1996;334:209–15. 10.1056/NEJM199601253340401. 8531996

[43] Reysenbach AL, Giver LJ, Wickham GS, Pace NR. Differential amplification of rRNA genes by polymerase chain reaction. Appl Environ Microbiol. 1992;58:3417–8. 10.1128/aem.58.10.3417-3418.1992. 1280061 PMC183115

[44] Parola P, Roux V, Camicas JL, Baradji I, Brouqui P, Raoult D. Detection of *Ehrlichiae* in African ticks by polymerase chain reaction. Trans R Soc Trop Med Hyg. 2000;94:707–8. 10.1016/S0035-9203(00)90243-8. 11198664

[45] Gubbels JM, de Vos AP, van der Weide M, Viseras J, Schouls LM, de Vries E, et al. Simultaneous detection of bovine *Theileria* and *Babesia* species by reverse line blot hybridization. J Clin Microbiol. 1999;37:1782–9. 10.1128/JCM.37.6.1782-1789. 10325324 PMC84950

[46] Kearse M, Moir R, Wilson A, Stones-Havas S, Cheung M, Sturrock S, et al. Geneious Basic: an integrated and extendable desktop software platform for the organization and analysis of sequence data. Bioinformatics. 2012;28:1647–9. 10.1093/bioinformatics/bts199. 22543367 PMC3371832

[47] Altschul SF, Gish W, Miller W, Myers EW, Lipman DJ. Basic local alignment search tool. J Mol Biol. 1990;215:403–10. 10.1016/S0022-2836(05)80360-2. 2231712

[48] Guindon S, Dufayard JF, Lefort V, Anisimova M, Hordijk W, Gascuel O. New algorithms and methods to estimate maximum-likelihood phylogenies: assessing the performance of PhyML 3.0. Syst Bio. 2010;59:307–21. 20525638 10.1093/sysbio/syq010

[49] Lefort V, Longueville JE, Gascuel O. SMS: Smart Model Selection in PhyML. Mol Biol Evol. 2017;34:2422–4. 10.1093/molbev/msx149. 28472384 PMC5850602

[50] Hall H, Bilsborrow J, David J, Dizkirici A, Könyves K, Culham A. Phylogenomic incongruence gives new perspectives on the taxonomic complexity of Muscari sl (Asparagaceae, Scilloideae). Taxon. 2025;74:1351–71.

[51] Hoffmann C, Stockhausen M, Merkel K, Calvignac-Spencer S, Leendertz FH. Assessing the feasibility of fly based surveillance of wildlife infectious diseases. Sci Rep. 2016;6:37952. 10.1038/srep37952. 27901062 PMC5128827

[52] Valente A, Jiolle D, Ravel S, Porciani A, Vial L, Michaud V, et al. Flying syringes for emerging enzootic virus screening: proof of concept for the development of noninvasive xenosurveillance tools based on tsetse flies. Transbound Emerg Dis. 2023;2023:9145289. 10.1155/2023/9145289. 40303700 PMC12016974

[53] Olagunju EA, Ayewumi IT, Adeleye BE. Effects of livestock-keeping on the transmission of mosquito-borne diseases. Zoonoses (Basel). 2024;4:e2024–36. 10.15212/ZOONOSES-2024-0036.

[54] Gerken KN, Olubowa RR, Chiuya T, Korir M, Fèvre EM, Stringer A, et al. Seasonal variation in mosquito abundance and environmental predictors in semi-pastoral southern Kenya: implications for endemic Rift Valley fever. Parasites Vectors. 2025;19:1. 10.1186/s13071-025-07122-1. 41310772 PMC12764129

[55] Zablon JO, Goel V, Giesbrecht D, Kariuki L, Mbogo C, Goedel WC, et al. Mapping mosquito diversity in Kenya correlating species distribution with malaria prevalence across varied climatic parameters. Malar J. 2026;25:76. 10.1186/s12936-025-05713-y. 41491483 PMC12870289

[56] Kocan KM, de la Fuente J, Blouin EF, Coetzee JF, Ewing SA. The natural history of *Anaplasma marginale*. Vet Parasitol. 2010;167:95–107. 10.1016/j.vetpar.2009.09.012. 19811876

[57] Sakamoto JM, Azad AF. Propagation of arthropod-borne *Rickettsia* spp. in two mosquito cell lines. Appl Environ Microbiol. 2007;73:6637–43. 10.1128/AEM.00923-07. 17766452 PMC2075076

[58] Mazzola V, Amerault TE, Roby TO. Electron microscope studies of *Anaplasma marginale* in an *Aedes albopictus* culture system. Am J Vet Res. 1979;40:1812–5. 525907

[59] Mwaki DM, Kidambasi KO, Kinyua J, Ogila K, Kigen C, Getange D, et al. Molecular detection of novel *Anaplasma* sp. and zoonotic hemopathogens in livestock and their hematophagous biting keds (genus *Hippobosca*) from Laisamis, northern Kenya. Open Research Africa. 2022;5:23. 10.12688/openresafrica.13404.1. 37396343 PMC10314185

[60] Welinder-Olsson C, Kjellin E, Vaht K, Jacobsson S, Wennerås C. First case of human 'Candidatus Neoehrlichia mikurensis’ infection in a febrile patient with chronic lymphocytic leukemia. J Clin Microbiol. 2010;48:1956–9. 10.1128/JCM.02423-09. 20220155 PMC2863919

[61] Vayssier-Taussat M, Le Rhun D, Buffet JP, Maaoui N, Galan M, Guivier E, et al. Candidatus Neoehrlichia mikurensis in bank voles. France Emerg Infect Dis. 2012;18:2063–5. 10.3201/eid1812.120846. 23171720 PMC3557860

[62] Tołkacz K, Kowalec M, Alsarraf M, Grzybek M, Dwużnik-Szarek D, Behnke JM, et al. Candidatus Neoehrlichia mikurensis and *Hepatozoon* sp. in voles (*Microtus* spp.): occurrence and evidence for vertical transmission. Sci Rep. 2023;13:1733. 10.1038/s41598-023-28346-0. 36720952 PMC9889374

[63] Palomar AM, García-Álvarez L, Santibáñez S, Portillo A, Oteo JA. Detection of tick-borne 'Candidatus Neoehrlichia mikurensis’ and *Anaplasma phagocytophilum* in Spain in 2013. Parasit Vectors. 2014;7:57. 10.1186/1756-3305-7-57. 24484637 PMC3912351

[64] Kamani J, Baneth G, Mumcuoglu KY, Waziri NE, Eyal O, Guthmann Y, et al. Molecular detection and characterization of tick-borne pathogens in dogs and ticks from Nigeria. PLoS Negl Trop Dis. 2013;7:e2108. 10.1371/journal.pntd.0002108. 23505591 PMC3591325

[65] Portillo A, Santibáñez P, Palomar AM, Santibáñez S, Oteo JA. Candidatus Neoehrlichia mikurensis. Europe New Microbes New Infect. 2018;22:30–6. 10.1016/j.nmni.2017.12.011. 29556406 PMC5857181

[66] Fehr JS, Bloemberg GV, Ritter C, Hombach M, Lüscher TF, Weber R, et al. Septicemia caused by tick-borne bacterial pathogen Candidatus Neoehrlichia mikurensis. Emerg Infect Dis. 2010;16:1127–9. 10.3201/eid1607.091907. 20587186 PMC3358111

[67] von Loewenich FD, Geissdörfer W, Disqué C, Matten J, Schett G, Sakka SG, et al. Detection of 'Candidatus Neoehrlichia mikurensis’ in two patients with severe febrile illnesses: evidence for a European sequence variant. J Clin Microbiol. 2010;48:2630–5. 10.1128/JCM.00588-10. 20519481 PMC2897504

[68] Quarsten H, Grankvist A, Høyvoll L, Myre IB, Skarpaas T, Kjelland V, et al. Candidatus Neoehrlichia mikurensis and *Borrelia burgdorferi* sensu lato detected in the blood of Norwegian patients with erythema migrans. Ticks Tick Borne Dis. 2017;8:715–20. 10.1016/j.ttbdis.2017.05.004. 28539197

[69] González-Carmona P, Portillo A, Cervera-Acedo C, González-Fernández D, Oteo JA. Candidatus Neoehrlichia mikurensis infection in patient with antecedent hematologic neoplasm. Spain Emerg Infect Dis. 2023;29:1659–62. 10.3201/eid2908.230428. 37486220 PMC10370848

[70] Labbé Sandelin L, Olofsson J, Tolf C, Rohlén L, Brudin L, Tjernberg I, et al. Detection of *Neoehrlichia mikurensis* DNA in blood donors in southeastern Sweden. Infect Dis (Lond). 2022;54:748–59. 10.1080/23744235.2022.2087732. 35724266

[71] Mens H, Gynthersen R, Christensen JR, Blinkenberg M, Sellebjerg F, El Fassi D, et al. Prevalence of tick-borne *Neoehrlichia mikurensis* in individuals undergoing B-cell depleting therapy in Denmark: a prospective cohort study 2023–2024. Int J Infect Dis. 2025;161:108069. 10.1016/j.ijid.2025.108069. 40972815

[72] Štefanić S, Grimm F, Mathis A, Winiger R, Verhulst NO. Xenosurveillance proof-of-principle: detection of *Toxoplasma gondii* and SARS-CoV-2 antibodies in mosquito blood meals by (pan)-specific ELISAs. Curr Res Parasitol Vector Borne Dis. 2022;2:100076. 10.1016/j.crpvbd.2022.100076. 36589872 PMC9795339

[73] Bitome-Essono PY, Ollomo B, Arnathau C, Durand P, Mokoudoum ND, Yacka-Mouele L, et al. Tracking zoonotic pathogens using blood-sucking flies as “flying syringes.” Elife. 2017;6:e22069. 10.7554/eLife.22069. 28347401 PMC5426900

[74] Mwakasungula S, Rougeron V, Arnathau C, Boundenga L, Miguel E, Boissière A, et al. Using haematophagous fly blood meals to study the diversity of blood-borne pathogens infecting wild mammals. Mol Ecol Resour. 2022;22:2915–27. 10.1111/1755-0998.13670. 35730337 PMC9796008

